# Retrograde ureteroscopic intrarenal surgery for large (1.6-3.5 cm) upper ureteric/renal calculus

**DOI:** 10.4103/0970-1591.60443

**Published:** 2010

**Authors:** M. Prabhakar

**Affiliations:** Department of Urology, Kalyani Urology Clinic, 104, Sampath Nagar Main Road, Opp Kongu Kalai Arangam, Erode - 638 011, Tamil Nadu, India

**Keywords:** Kidney stone treatment, laser ureteroscopy, RIRS

## Abstract

**Objective:**

To assess the feasibility of retrograde ureteroscopic intrarenal surgery (RIRS) as a viable alternate to percutaneous nephrostolithotripsy (PCNL) in treating patients with renal and upper ureteric calculus of 1.6 cm to 3.5 cm stone burden.

**Materials and Methods:**

From October 2007 to November 2008, a total of 30 cases of upper ureteric and renal stone of 1.6 cm to 3.5 cm (Average size 2.5 cm) stone burden, for which PCNL would be done otherwise, were treated by RIRS with combined flexible and semi rigid ureteroscope and stones fragmented with holmium laser. The patients were discharged after 24 hours of the procedure and allowed to resume normal work after two days. X ray KUB for radio opaque stones and ultrasound for all the cases were done after three weeks and if any residual fragments of any size were present the patient was taken up for re-look flexible ureteroscopy under anesthesia. Stent and residual fragments were removed. If there was no residue the stent was removed under local anesthesia.

**Results:**

Complete clearance was considered if there were no fragments on USG screening after three weeks. Twenty six (86.6%) patients out of 30 had complete clearance in the first sitting and 4 (13.3%) patients needed re-look flexible ureteroscopy. The stone free rate in RIRS is 86.6% in the first sitting and 100% at second sitting.

**Conclusion:**

RIRS is superior in terms of less complication, less morbidity and good stone free rate and has an advantage of one day of hospital stay and resuming duties after two days. RIRS is the best option for managing extracorporeal shockwave lithotripsy failed and post PCNL residual calculus. RIRS is definitely a viable alternate for PCNL for upper tract stones up to 3.5 cm.

## INTRODUCTION

Flexible ureteroscopy was initially used only for diagnostic purpose as there was no working channel in the older models. However, with the advent of new generation miniaturized flexible ureteroscopes with better optics, improved deflection mechanism and wide rage of accessory instruments like, tipless nitinol baskets, double floppy tip guide wire, thinner hydrophilic coated kink resistant access sheath and good irrigation pumps and good fragmentation devices like Holmium laser with thinner fibers (200 micron) to access lower calyx without affecting the deflection of the flexible scope the indications for the use of flexible scopes have widened to a variety of procedures like treatment of kidney stones, renal pelvic tumors and calyceal diverticulum's.

percutaneous nephrostolithotripsy (PCNL) is a gold standard procedure for large kidney stones with a potential morbidity of bleeding, which might need angioembolization, and also has certain limitations in patients with bleeding diathesis,[1] obesity and malrotated kidneys. Retrograde ureteroscopic intrarenal surgery (RIRS) is a less morbid procedure than PCNL. The usage of RIRS is presently limited to patients who are contraindicated for PCNL/shockwave lithotripsy (SWL) like bleeding diathesis, morbid obesity, malrotated/malpositioned kidney, horse shoe kidney, and calculus (<1.5 cm) in unfavorable lower calyx.

The technical developments in laser technology and significant improvement in flexible ureteroscopes have made RIRS for larger ureteric/renal stones possible. The low complication rate gives RIRS for ureteric/renal stones superiority over the invasive percutaneous approach, which is associated with significant morbidity, even in experienced hands.

So, we have evaluated the feasibility of RIRS as a viable alternate to PCNL in treating patients with renal and upper ureteric calculus of 1.6 cm to 3.5 cm stone burden, the cases which are usually taken up for PCNL otherwise.

## MATERIALS AND METHODS

From October 2007 to November 2008, a total of 30 cases of upper ureteric and renal stone of 1.6 cm to 3.5 cm (Average size 2.5 cm) stone burden for which PCNL would be done otherwise were treated by RIRS.

The stone size was measured on a plain X ray KUB and the longest diameter of the stone was measured. The stone burden was calculated by adding up all the stones in the upper ureter and the kidney. Ultrasound was used to measure the size of radiolucent stones. All the patients were worked up with an IVP, routine blood and urine investigation and treated as out patient with appropriate antibiotics if the urine culture was positive. The patients were admitted the previous day evening of the procedure and they underwent this procedure under spinal anesthesia and converted to general anesthesia if required.

We do not routinely pre stent the patient. All the patients underwent ureteric orifice dilatation with a 6/12 Nottingham dilator. RGP was done in all cases to understand the calyceal anatomy. Ureteroscopy with a semi rigid ureteroscope (wolf 7/8.5 F) was done in all cases. Upper ureteric, renal pelvic or upper calyx calculi, if easily accessible, are fragmented with Holmium: YAG laser (Dornier Medilas H 20). A both end floppy tip guide wire (COOK) was inserted under the C arm guidance into the semi rigid URS and placed in the renal pelvis. An access sheath kink-resistant and hydrophilic coated (cook inner diameter-12 F, outer diameter 13.5 F, 35 cm-FLEXOR) is passed over the guide wire under C arm guidance up to the PUJ. If the access sheath cannot be passed due to tight ureter the flexible ureteroscope (ACMI Dura - 8) is back loaded over the guide wire and the scope was negotiated into the ureter up to the renal pelvis and then the guide wire is removed. Irrigation pressure pump is always used to keep the field clear.

Stones from the calyx are repositioned into the upper calyx with the help of a (2.2 F Cook) O-tip basket, this step helps in increasing the life of the flexible ureteroscope. If the stone is big and not basketable, a 270 micron fiber is used to fragment the calyceal stone into two or three pieces. The fragments are repositioned into the upper calyx and the stones in the upper calyx are fragmented with 400 micron laser fiber [8.4 watts (1.2 joules and 7 hertz) for soft stones and 9.1 watts (1.3 joules and 7 hertz) for hard stones]. Small stones are basketed out with O Tip basket.

Three methods were used to fragment the stones:

- Painting method - The laser fiber was moved over the stone just like painting with a brush, this method was used in the case of soft stones.
- Drilling method - Multiple drills were made over the stone and then the intermittent ridge was fragmented to make it into small bits.
- Popcorn effect - This method was used to break large fragments into tiny bits; the laser was fired in the middle of the large fragments with a distance of about 5 mm without focusing on any particular fragment. The energy was not changed but the frequency was increased to 9-10 hertz. This causes the fragments to fly like popcorn and in this process the stones get hit by the laser fiber and become tiny fragments. This method helps to make the fragments into tiny bits, which are allowed to be passed out in urine. It saves a lot of time when compared to breaking individual fragments. The free-flying of the fragments with the irrigation fluid indicates that the fragments are sufficiently small to be passed out in the urine.

The largest fragment was basketed out to assess the size. Contrast was injected in the working port of the flexible scope and all the calyces were inspected both with direct vision through the flexible ureteroscope and C arm to be sure that no large fragments were left out in any calyx. Double J Stenting was routinely done in all cases. The patient was discharged after 24 hours of the procedure and allowed to resume normal work after two days. X ray KUB for radio opaque stones and Ultrasound for all the cases were done after three weeks and if any residual fragments of any size were present the patient was taken up for re-look flexible ureteroscopy under anesthesia. Stent and residual fragments were removed. If there was no residue the stent was removed under local anesthesia.

## RESULTS

Stone size ranged from 1.6 cm to 3.5 cm (average size-2.5 cm). Ten cases with solitary stones (size 1.6-2.2 cm, average-1.9 cm). 20 cases with multiple stones. Four patients with ESWL failure (three patients with two sittings and one patient with one sitting) and one patient with a post PCNL residue were treated.

Operating time was 45 minutes to 190 minutes (average time 92 minutes) the time was calculated from starting the endoscopic procedure till catheterization. Anesthesia, positioning and preparation time were not included. Complete clearance was considered if there were no fragments on USG screening after 3 weeks. Twenty six (86.6%) patients out of 30 had complete clearance in the first sitting and 4 (13.3%) patients needed re-look flexible ureteroscopy. All four patients had residual fragments less that 6 mm which needed only basketing; there was no need for fragmentation.

Five out of 30 patients had stent related complaints like dysuria, flank pain during urination and mild hematuria which settled with increased fluid intake and analgesics. One patient developed post-spinal headache which settled with bed rest, increased fluid intake and analgesics.

All the patients were discharged after 24 hrs of the procedure; 29 out of 30 patients could resume normal work after two days of the procedure. One patient resumed normal work after seven days due to postspinal headache.

## DISCUSSION

PCNL was the only option to treat large upper ureteric/renal stones before the introduction of RIRS. Huffman and associates[2] first reported the use of ureteroscopy to treat renal pelvic calculus in 1983. Grasso and associates[3] have shown the use of RIRS for large renal stones in patients who had comorbid conditions and were not fit for PCNL. Some authors have proposed a combination of ureteroscopy with SWL as the management alternative to PCNL.[4,5]

Several techniques can be applied to improve the fragmentation and removal of large upper ureteric and renal calculus by RIRS and minimize the need for re-look surgery. The major time consuming maneuver in RIRS is trying to fragment the stone in lower or middle calyx. This can be overcome by repositioning the stone in a favorable upper calyx. This will help the flexible scope to be straight during fragmentation process and avoids strain on the deflection mechanism and the risk of laser fiber damaging the scope.

The second method of reducing the operating time is by using the popcorn method. All the fragments are placed in a single calyx and the laser fiber fired at the middle of the fragments with out focusing a particular fragment, this saves a lot of time and breaks the stones in to size <4 mm which is sufficient to be passed out in the urine. The bulk of residual fragments are considerably less when laser is used to fragment,[6,7] as compared with pneumatic lithotripter, because the laser vaporizes most of the stones and the dust is washed out in the flowing saline during the procedure. Continuous flow pressure pump is helpful to keep the vision clear through out the procedure which also helps reduce the operative time.

Previous studies have addressed the issue of primary RIRS for kidney stones 1-2 cm size (Ave 1.25 cm).[8] They have retrospectively analyzed and compared RIRS with PCNL. They have a stone-free rate of 67% in RIRS group as compared to 87% in PCNL group. RIRS was done as outpatient and PCNL had an average of two days of hospital stay. The complication rate was nil in RIRS group whereas, 13% in PCNL group.

Sofer *et al*.,[9] did a retrospective analysis of 598 patients with upper tract calculi with mean size of 13.5 mm and achieved an over all stone free rate of 84% for renal calculi. Grasso *et al*.,[3] treated renal stones 2 cm or greater with RIRS, for patients who had comorbid conditions and in whom PCNL was not possible, and achieved an over all stone free rate of 93% in renal and 100% in upper ureteric calculus.

In a retrospective analysis on 23 patients, selected for RIRS instead of PCNL due to comorbidity, obesity, anatomical problems in kidney and previous treatment failure, the overall stone free rate was 74%.[10] They stratified the locations within the kidney with the stone free rate for lower pole and locations other than the lower pole which was 83% and 74% respectively, showing that the lower pole stones had a better stone free rate. The linear calculated diameter was inversely propositional to the stone free rate (10-20 mm-100%, 20-30 mm-87.5%, 30-40 mm-60%, >40 mm-40%)

Jason *et al*.,[11] performed combined RIRS with SWL in same sitting for 14 patients who were advised PCNL and the patients were either unfit or not willing for PCNL. The mean calculated stone surface area was 847 mm^2^ (Range 58 mm^2^-1850 mm^2^). 14% of the patients were stone free after first sitting and over all stone free rate was 77%.

In our study, we have found a stone free rate of 86.6% in first sitting and 100% in the second sitting. No major complications were reported. All the patients were discharged in 24 hrs.96.6% (29/30) of patients resumed normal duties on the third postoperative day.

All four patients with ESWL failure and one patient with post PCNL residue were completely cleared of the stone in the first sitting. RIRS for stone >1.5 cm have either poor results as compared with PCNL or are done as staged procedure with multiple admissions which makes it a non cost effective option. We have refined our technique by combining the use of flexible ureteroscope, semi rigid ureteroscope, ‘O’ tip baskets. To achieve a better stone free rate as compared to other studies, less number of hospital stay and least complications than PCNL.

This study was not done in a randomized way and did not have a control group. The follow-up for residual fragments was done with ultrasound and X-ray KUB. A future study with prospective double blind randomized trial comparing RIRS and PCNL and the follow-up with NCCT would give even more accurate data to conclude RIRS as an alternative option for PCNL.

## CONCLUSION

The stone free rate in RIRS is 86.6% in first sitting and 100% with second sitting. RIRS is superior in terms of less complication, less morbidity and good stone free rate and has an advantage of one day of hospital stay and resuming duties after two days. RIRS is the best option for managing ESWL failed and post PCNL residual calculus. RIRS is definitely a viable alternate for PCNL for upper tract stones up to 3.5 cm.
